# Hospital and Institutional News

**Published:** 1914-10-03

**Authors:** 


### HOSPITAL AND INSTITUTIONAL NEWS.

### "THE HOSPITAL'S" SPECIAL NUMBER.

In view of the hospital arrangements necessitated by the outbreak of war, and of the amount of emergency hospital work which is being done in the country, we have decided to devote our customary Autumn Special Number to an account of the practical problems presented by this work. Naturally, difficulties in the way of securing expert assistance at a time when all possessed of it are devoting their time to practical administration have been great, but we shall hope next week, nevertheless, to present information on a number of practical points, illustrating the cost, requirements, and equipment of emergency hospitals. In addition, we hope to publish a full account of a Territorial base hospital from the same point of view. The effort to collect information on the practical points presented for solution by the hospitals during the present crisis will, we hope, make our Special Number of lasting value.

### BEDSTEAD LIFTERS COMPETITION.

The proposed competition announced in The Hospital of May 23 and succeeding issues, owing probably to the holiday season, did not produce the minimum number of competitors whose papers conformed with the conditions. The competition could not, therefore, be organised for the time at any rate. Several of the intending competitors, instead of dealing with bedstead lifters, the articles to which the competition was limited, sent in descriptions of all sorts of appliances connected with the lifting of patients, with which latter the proposed competition had no connection. Our hope of a practical competition, therefore, has not been fulfilled. We hope, however in the autumn that sufficient interest in the subject may be manifest to enable a competition to be organised, for an efficient and simple bedstead lifter of the best practical design which would meet a felt want in many institutions.

### HUT HOSPITALS: A RED CROSS SUGGESTION.

Under this title the *Spectator* of the 26th ult. raises the question as to what provision will be made for some 490,000 men (recruits) in four or five camps in various parts of the country for looking after the sick, each of which camps will require a good deal of hospital accommodation. On inquiry we find that the requisite hut hospital accommodation with an adequate medical equipment will be provided in the case of each of these camps, so that there will be neither opportunity nor necessity to enlist the services of the Voluntary Aid Detachments and others as suggested by our contemporary.

### NEW SECRETARY AT NORTH ORMESBY HOSPITAL

In succession to Mr. E. C. Smith, who has taken up the appointment of secretary to the Queen's Hospital at Birmingham, as already announced in The Hospital, Mr. Arthur Williams, who has been assistant secretary-superintendent to the Swansea General and Eye Hospital for the last 6½ years, has been appointed secretary to the North Ormesby Hospital. It is interesting to note that Mr. Williams was appointed to Swansea Hospital as Mr. Smith's successor, when he took up his duties at Middlesborough, so the committee have paid both a great compliment in appointing him as the new secretary to North Ormesby Hospital.

### A CURIOUS PUBLIC MISCONCEPTION.

One of the most curious misconceptions created by the war is that of which several great provincial hospitals have complained, that the public in the localities which they serve is under the impression that little is being done by the hospital for the wounded soldiers. So far from this being the case, of course, we have already been compelled to emphasise the duties of the voluntary hospitals to the civil population. Therefore, it is specially hard that, in view of this warning, the hospitals should be open to misconception on the part of their supporters. Instances of this, however, have been provided in the case of the Royal Infirmary, Sheffield, and the Royal Victoria Infirmary, Newcastle. Since our own columns have provided such striking and continuous evidence of the work done for the soldiers by both these institutions, we may well refer to Sir George Hare Philipson's reminder at the quarterly court of the Newcastle institution only last Saturday. He pointed out that the Victoria Infirmary was working in combination with the military authorities at Armstrong College, alluded to the new temporary wards, which we have already described, and gave high praise to Dr. G. H. Hume, as chairman of the emergency committee, for having raised the necessary funds, amounting to £2,000, for the provision of the temporary wards referred to. At present there are 300 civilians and eighty-two soldiers in the Victoria Infirmary, and the school and charities committee have placed forty beds in the Home for Incurables at the disposal of the infirmary for convalescent patients. These facts, we hope, should prove sufficient to disabuse the public mind of what is surely the most curious and undeserved reproach which the voluntary hospitals have ever had to suffer. We reproduce its refutation at Newcastle as an example to other institutions, similarly misunderstood, to give a public account of the double work which they are so generously performing on behalf of both the military and the civil population.

### PUBLIC DEPARTMENTS AND MEDICAL ETIQUETTE.

One of the troubles all institutional medical officers have to encounter is the difficulty of explaining to employers that they cannot be given information as to the nature of the illness of their employees without the consent of the patient concerned. One is not very much surprised when private employers protest against this elementary rule of professional secrecy, because one can make allowances for their ignorance of the confidential position in which a medical man stands as regards his patients. The case is, however, different when the medical officer of a public body, who ought to know better, makes a similar protest. An instance occurred recently at a London Poor-Law infirmary. The medical department of the Post Office wrote asking for a certificate of the nature of the illness of a postman who was an inmate of the infirmary. The medical superintendent naturally replied that such a certificate could only be given to the patient from whom it might be obtained. One would imagine that no medical man could expect or desire any other reply. This was not, however, the view taken by the medical department of the Post Office, and a letter was sent protesting against the enormity of a public official refusing to supply the fullest information about the illness of a man employed by a public body to his employers. Poor-Law medical officers do not require to be told that the ordinary rules of professional etiquette are not to be violated in the interests of a public body any more than in those of a private employer. It is open to every employer, whether private or public, to obtain any medical certificate required through the patient, and it is for the latter and for the latter only to decide whether such information shall be given.

### THE INFIRMARY QUESTION AT BATH.

The Bath Board of Guardians have again taken occasion to postpone provision of the new infirmary, the need for which, on unimpeachable medical and official authority, has been emphasised so often in The Hospital. At a meeting of the Guardians last week Mr. Wills moved the adoption of the report of the joint general purposes and house committees, which in view of the European crisis had resolved that the matter be postponed for the present, but that steps be taken at once for temporarily removing the defects of the heating of the infirmary. This motion, which was seconded by the Chairman, Colonel Clayton, was ultimately withdrawn in favour of an amendment instructing Mr. Malcolm Evans, the engineer, to prepare plans for the heating and alterations at Frome Road House, " it being understood that a separate hospital may be built at a favourable opportunity." Mr. Curd protested against nothing being done to remedy the defects pointed out by their medical officer and accused the board of continually shirking their responsibility in the matter, pointing his criticism with the remark that if new heating arrangements were put in they would not get a new infirmary. The plea of the joint committee that the European crisis must occasion further postponement of a work already notoriously overdue seems to us particularly unfortunate, for it has been the public policy of statesmen and Government Departments at the present time, so far from cancelling building schemes or curtailing new departures, to press forward building operations and to undertake at once work which in peaceful times would not be thought necessary to hasten. This being the case, we consider that the Bath Guardians have even less shred of excuse for not building a new infirmary than ever, and that their latest excuse has cut the ground from under their feet, and, apart from the infirmary question in itself, convicted them of a grave want of public spirit in a time when Boards of Guardians, more perhaps than any other local authority, are being expected to show it.

### A DOCTOR'S CHARGES AGAINST A HOSPITAL.

An unfortunate state of tension has arisen in connection with the Wellingborough Cottage Hospital between the committee, a local practitioner, Dr. Waters, and the Medical Institute. It arose out of a letter which Dr. Waters sent to the local papers alleging, in effect, that the same treatment was not given to him, in regard to the admission of patients, by the committee as was accorded to other medical men of the town. A special meeting of the hospital committee was at once called to consider Dr. Waters' letter, at which questions arising out of the admission of recent patients recommended by him were considered, and the result was the passing of a resolution stating that the committee, having heard " Dr. Waters' statement, consider his charges utterly baseless." Since, moreover, several members of the committee considered that Dr. Waters should either apologise or be asked to resign, it was agreed that this question should be adjourned till the next meeting of the committee, at which the executive of the Medical Institute should be invited to attend. This adjourned meeting of the hospital committee was held last week, at which Mr. George Henson presided. A letter was read from Dr. Waters, in the course of which he withdrew his letter (to the papers) and expressed his regret for any pain caused by the position he had taken. The Chairman voiced the opinion of the meeting that Dr. Waters' letter did not go far enough, and a form of apology was drafted for him to sign and publish in the local papers, failing which it was resolved that he should be struck off the medical staff. Mr. Thomas Maddock, hon. secretary, read two letters from the Medical Institute. The first expressed regret at Dr. Waters' letter to the press, and the second protested against the statements made at the previous meeting to the effect that no direct subscriptions were received from the Foresters, 1,600 of whom are represented by the institute. The institute, indeed, a day or two later has defined its attitude by stating that, while it does not approve or support Dr. Waters' letter, considering he has already withdrawn it, it cannot therefore, recommend him to sign the " humiliating proposal " presented to him, and asks the hospital committee at once to take his name off the hospital staff. Presumably this will be done, as the want of tact which has characterised all parties to the dispute virtually leaves no other course open. It is to be regretted that the medical institute has been drawn at all into a matter which should have been confined to Dr. Waters and the hospital committee; but the moral is chiefly for the doctor, who should learn the lesson taught by public life, that if you are going to withdraw a public accusation at all, you had better do so handsomely.

### AN ADMINISTRATIVE DILEMMA AT COVENTRY.

The house committee of the Coventry and Warwickshire Hospital having reported a decision to increase the number of beds offered to the military authorities from twenty to thirty and its acceptance by the War Office, a difficulty has arisen over a waiting-list of twenty while there are still in the hospital unoccupied beds. At the recent monthly meeting Mr. A. J. Johnston raised the matter, and Mr. E. M. Iliffe declared that the hospital should be carried on as usual, and that there should be no waiting-list at all. If there were no waiting-list the hospital would be in a far better position when a rush from the War Office occurred. Therefore he and Mr. H. Twist, the vice-chairman, were going to inquire why twenty patients were waiting admission, in spite of the unoccupied beds. Dr. Webb Fowler remarked that, having had their offer of beds accepted by the War Office, they must be prepared for some inconvenience. Mr. Iliffe, however, is quite right in insisting on the hospital's duty to the civil population, and unless arrangements can be made to deal with serious waiting cases, it seems undesirable to increase the number of beds reserved for the wounded.

### PANEL PRACTITIONERS AND INCAPACITY DUE TO PREGNANCY.

The circular to approved societies, recently issued by the Insurance Commissioners, stating that no distinction should be drawn as regards the payment of sickness or disablement benefit between incapacity due to pregnancy and incapacity due to other causes, has now been followed by a memorandum to medical practitioners on the same subject. By it panel doctors are informed that where they are satisfied of incapacity "a certificate should not be withheld on the ground that the incapacity is due to or accompanied by pregnancy." The memorandum points out that since a special grant has been provided in respect of the payment of claims for sickness benefit arising out of pregnancy, it is essential from the point of view of the societies who may claim a share of the grant that "where the incapacity for work is either directly or indirectly due to pregnancy, that fact should be clearly indicated on the certificate." The criterion of incapacity, on which alone the grant of a certificate depends, is, of course, not affected by this change of practice, and the result is merely that incapacity accompanied by pregnancy is placed on the same footing, as regards sickness benefit, as any other incapacity, and that, in view of the special grant for claims arising out of pregnancy, this cause, whenever it occurs, must be entered on the certificate by the practitioner.

### THE NEW PHARMACOPŒIA.

The British Pharmacopœia, 1914, advance copies of which were placed for inspection at the office of the General Medical Council on October 1, does not differ very much in appearance from the Pharmacopœia which has been official since 1898, and, as a matter of fact, it seems, at first sight, to have undergone very few changes of a revolutionary character. The number of omissions vastly exceeds the number of additions, and surprisingly few of the newer synthetic drugs of German origin have received official sanction. It is not possible this week to go fully into the details of the changes, and it must suffice to state that apart from the omissions and additions the main alterations are as follows: The metric system has been employed for all pharmaceutical computations. This system has also been employed for the specification of doses, in the expectation that in the near future the system will generally be adopted by British prescribers, but as a transitional provision doses have also been expressed in terms of the imperial system. The number of crude drugs and their preparations which are required to contain a definite proportion of the chief active constituents has been increased. The recommendations of the International Congress on potent preparations have been adopted, and hence the strengths of a number of compounds have been altered considerably. It has also been found desirable to indicate a limit to the proportion of lead or of arsenic permissibly present as an impurity in many pharmacopœial substances. A detailed review is, of course, not possible in this issue, but the above indicates to some extent the main changes.

### ULSTER HOSPITAL FOR SERVICE IN FRANCE.

United Ireland is manifesting a fine spirit of assistance in the war, but we have to remember that this help has not only consisted in the provision of troops and Volunteers. The military organisation of the latter in Ulster and elsewhere was also, as we noted at the time, accompanied by extensive hospital preparations, with the result that the offer of the North Tyrone Ulster Volunteer Force Hospital has now been accepted by the French Government. The local staff of medical men and nurses will accompany the hospital, which, as soon as its complete equipment has been arranged for, will leave for service at Pau. When established there our ally will be responsible for its maintenance, but the cost of equipment and of transport will be arranged for by the donors. The St. John Ambulance Association are helping with the work. However extensive the hospital preparations in Ulster were when the possibility of disturbance was threatened, it is of course one thing to prepare for wounded and another to transport an equipped hospital for service with the troops abroad. We can only say that, apart from wishing the hospital every success, we hope that the elaborate lessons in preparation and the practical test of transport will provide experience to be treasured in Ulster when peace once more removes the stimulus to preparations of this kind.

### WIVES OF RECRUITS AND MATERNITY BENEFIT.

How difficult it is for the nation to undertake satisfactorily their paramount duty to the dependants of both the Services without taint of charity, patronage, or relief our previous *exposé* of the first attempts in this direction have abundantly shown. But we regret to say that we are not yet out of the wood, and that in some cases red tape is complained of as depriving insured women, whose husbands are away on active service, of maternity benefit, at least for a time. The tragic irony of the situation arises from the regulations laid down by the Government with a view to helping the dependants of recruits to the prompt receipt of the Government allowances which are their due. Complaints in fact are made that the wives of men who have enlisted are unable to obtain maternity benefit, because they cannot produce their marriage certificates. These certificates, of course, as we pointed out a fortnight ago, have now to be brought to the military authorities by each recruit, and are now at the War Office. The result is that cases of difficulty arise, because a certificate, like a person, cannot be in two places at once. It surely does not require much administrative faculty, however, to see that this difficulty is overcome with promptitude. Surely the recruit could be furnished with an official receipt to be given to his wife for presentation, if required, in lieu of the certificate? Indeed, there are a dozen simple expedients which might be adopted, and which, since the numbers involved are so large, should be quickly decided on, or the helping right hand of the State Insurance Act may be disabled by the clumsy efforts of the left to provide assistance in another direction. Truly at present it is a sad case of the right hand not knowing what the left hand has done.

### GUARDIANS AND MILITARY CAMPS.

Several Boards of Guardians having institutions in the neighbourhood of military camps have made arrangements which will add considerably to the comfort and well-being of the soldiers. At Steyning, in Sussex, for instance, the Guardians have authorised the master to allow the men from the camp in the district to use the dining-room of the workhouse for purposes of recreation after 7 p.m. As the dining-room is furnished with a piano, which is also to be placed at the men's disposal, this kind thought of the Guardians is likely to be greatly appreciated. The men are also to be allowed to use the bath-rooms. Similarly at Stanway, near Colchester, the Board-room is to be placed at the disposal of the soldiers billeted in the district, and the workhouse laundry has undertaken part of the washing of the men's clothes from the R.A.M.C. camp in the neighbourhood. These examples are commended to the attention of other Boards of Guardians having military camps near their institutions.

### ARMY AIRMEN AND THE WESTMINSTER GUARDIANS.

On request of the Director-General of the Medical Department of the Navy the Westminster Board of Guardians have agreed to accept cases of sick and wounded from the Royal Naval Flying Service at the Hendon Aviation Ground at their infirmary at Colindale Avenue, Hendon. The Admiralty do not expect that cases will be numerous, but they are anxious that a ward should be reserved for their use. The Director-General further asked the Guardians to mention a definite sum as a daily or weekly charge for each case under treatment. The proximity of the infirmary to the flying ground, apart altogether from patriotic motives, marks out the institution as peculiarly fitted for this purpose, and is only another instance of how far the modern Poor-Law infirmary has outgrown its constitutional function, and how nominal is the supposed qualification for admission to its wards. The Westminster Guardians' institution will, however, have the distinction of being the first Poor-Law infirmary to possess a special ward for airmen, and its occupants are, of course, likely to be suffering from the ordinary disabilities of humanity rather than from any disease occasioned by flying in war.

### LIVERPOOL DENTAL HOSPITAL AND THE WAR.

That every class of hospital is contributing to the war may be judged by the losses to the staff which its outbreak has caused to the Liverpool Dental Hospital. The acting treasurer, Mr. G. H. Cohen, has been called up, and his place is being taken by Professor D. J. Harvey-Gibson. Four members of the staff, Colonel L. J. Osborn, Captain J. W. Lloyd, Dr. F. W. Bailey, and Dr. G. R. F. Smith, have also been mobilised, and about half the students have joined the Colours. This has led to an invitation to twelve old students to assist in the treatment of recruits, for whose benefit a dental aid fund has been set on foot. The committee estimate that there are 3,000 Territorials in the neighbourhood requiring dental treatment, and up to last week over 500 have attended at the hospital. Indeed, the attendance has been so numerous that the hours of treatment have been extended to the afternoons, during which period recruits and Territorials alone are admitted. We trust that this prompt and practical extension of work will induce the public to rally to the support of the hospital, as well as to the dental aid fund, which ought to be well maintained since it comes to the assistance of those who have been rejected for service on account of their teeth.

### THE RECRUITS AND VACCINATION.

We have received a communication from the National Anti-Vaccination League drawing attention to a memorial issued by the League to the Secretary of State for War regarding the pressure now being put on Territorials to be vaccinated. The memorial includes certain questions and answers in Parliament to show that "there are no regulations enforcing vaccination on the Territorial Force." The contention of the League that the pressure being brought to bear on the men is having an adverse influence on recruiting, whether really an exaggeration or not, at least bears out the contention of our leading article last week that compulsion is not to be thought of. In view of the need for placing few stumbling-blocks in the way of recruits, the following extract from the League's letter which accompanies the circular may be quoted: "Bearing in mind the fact that commanding officers were instructed to respect the conscientious objections of the men, and were circularised to this effect as recently as August 23, and also that it was distinctly stated in 1908 that there are no regulations in existence compelling any members of the Territorial Force to be vaccinated, the pressure which has been exercised in this matter is most unfair." It is surely most inadvisable, while there is no objection to the educational campaign by medical officers being undertaken on the lines which we have laid down.

### THE OBSTRUCTION OF THE BROMLEY GUARDIANS.

The need of new infirmary accommodation in connection with the workhouse of the Bromley Guardians is still being made the occasion for the appointment of committees to consider different points concerning it. At a meeting of the Guardians last week, presided over by Alderman A. T. Waring, another report was presented and, after much discussion, another committee appointed to report upon the report. The matter before the meeting was the report of the architect, Mr. F. Danby Smith, containing the amended plans suggested in June at a conference between the Guardians and the Local Government Board, the result of which was a saving of £5,981 on the projected outlay. The minimum number of beds required for the new infirmary was accepted as 140, and, as Dr. James Allan remarked, this minimum had been passed by the Board. The Chairman, however, declared that much more information was necessary, and he therefore suggested that the architect's report should be referred to the committee who had gone into the matter before and that that committee should report upon each item in detail. Against this, Dr. Allan's reminder that the committee had met many times and gone into the details was the unavailing and solitary protest, and a committee was eventually elected. The only comment necessary is that the matter has already been discussed and reported on for two years or more, so last week's meeting has all the appearance of obstruction to a work which, apart from the intrinsic need for it, is just that type of building which Guardians and public bodies have been specially urged to undertake promptly at the present time.

### THE DEPLETED STAFFS OF GLASGOW HOSPITALS.

The large number of medical men who have left the staffs of the Glasgow institutions for active service have created a difficulty in the medical service which has led the health committee of the Glasgow Corporation to undertake special measures. They recommend, in brief, a scheme for the appointment of visiting physicians to act as consultants to the resident assistant medical men. Under the scheme two of these visiting physicians are to visit daily the institutions at Ruchill and Belvidere, as it is these hospitals especially where staffs have been depleted. The new scheme is directed primarily to their needs, but our readers will be interested to compare its provisions with those discussed in our leading article for remedying the analogous difficulty in the case of the medical staffs of the Poor-Law infirmaries. We are careful to record the Glasgow scheme because, as our leading article shows, there are right and wrong ways of meeting the situation created by the war, and the employment of visiting physicians is far preferable to other suggested expedients.

### THE SCHOOL OF PHARMACY AND THE WAR.

The School of Pharmacy of the Pharmaceutical Society, the Alma Mater of so many of the best-known of our hospital pharmacists, entered upon the seventy-third year of its existence on Wednesday last (September 30). Dr. Lauriston Shaw, who delivered the inaugural address, congratulated the authorities of the School on having been able at this period of national crisis to meet together for the purpose of carrying on their routine duties punctually at the accustomed time. He admitted that it must have been difficult for many to decide whether the path of duty lay in offering their services to their countries as combatants or in following the no less important function of preparing themselves for their profession at home. He went on to say that the full power of our Navy and Army could not possibly be maintained without an efficient Medical Service, including a pharmaceutical department. The men at the Front must know not only that their own medical needs would be dealt with as promptly as circumstances would allow, but must feel confident that those whom they had left behind and for whose homes they were fighting would receive such comforts as medical science could afford them when stricken down by illness. He was glad to learn that the authorities of the School of Pharmacy had made full provision that no actual or prospective student should suffer in any avoidable way by the interruption or postponement of his studies as a result of his engagement in military duties.

### WOMEN PHARMACISTS AT LONDON INFIRMARIES.

The Paddington Board of Guardians have had under consideration the question of a re-arrangement of the supply of medicines to the indoor and outdoor sick persons. Miss Constance Andrews has been appointed in charge, as chief pharmacist of the infirmary and also of the outdoor dispensary, and will have a qualified woman pharmacist as her assistant. Miss Andrews, who is a pharmaceutical chemist, was a student at the London College of Pharmacy before qualifying, also an official of the Women Pharmacists' Association and a member of the Public Pharmacists' and Dispensers' Association. Although there are several qualified women engaged as dispensers at hospitals and infirmaries, we understand this is the only Union in the Metropolitan area where a woman pharmacist is in complete charge of the arrangements for the supply of medicines.

### CARDIFF HOSPITAL AND SIR W. J. THOMAS.

Cardiff docksmen and other friends of Sir William James Thomas recently subscribed to a testimonial in honour of Sir William's knighthood. Some £1,600 was raised for the purpose, but Sir William declined any personal consideration, requesting that any subscription should go to the hospital. In the presence of a large and representative company, Mr. Daniel Radcliffe made the presentation at the Cardiff Exchange last week. It took the form of the endowment of a bed at King Edward VII.'s Hospital, Cardiff (1,000 guineas), and a cheque for £180 for the Royal *Hamadryad* Seamen's Hospital, whilst the docksmen had their wish gratified in securing a bronze bust of Sir William, by Sir Goscombe John, a striking likeness and a work of art, which is to find a permanent home in the hospital that owes so much to Sir William's munificence. It may be recalled that he has already personally endowed sixteen beds in King Edward VII.'s Hospital, so that the testimonial bed will make seventeen identified with his name. Sir William, in returning thanks to his friends for their generous gift, impressed upon them that they should make gifts to the hospitals in their lifetime, and witness the joy which resulted from such gifts. He regarded that testimonial as "a clap on the back" from the docksmen, who wanted him to go on with the work. He hoped soon to see the medical school built and equipped and doing its full share of work.

### POOR-LAW MEDICAL OFFICERS AND GERMAN PRISONERS.

At a recent meeting of the Council of the Poor-Law Medical Officers' Association of England and Wales, at which the increased work likely to be thrown upon the Poor-Law Medical Service was discussed, the question of the treatment of sickness among German prisoners was alluded to. As a result of the discussion it was decided that if Poor-Law medical officers were called upon to attend cases of sickness arising among German prisoners extra payment ought to be made to them. How far Poor-Law medical officers are likely to be called in to attend German prisoners is not at present clear, seeing that for the most part they have been passed on to the military or larger voluntary hospitals. Distinctions of any kind made by medical men in the treatment of wounded are invidious and to be deprecated, but in the case of prisoners not among wounded, but passed on to concentration camps or other centres in this country, the matter wears a different aspect.

### THE SUCCESS OF A CENTRAL DRUG STORE.

The value of the suggestion made in the columns of The Hospital on August 29, p. 592, of a central drug store for the Metropolitan Boards of Guardians, is seen in the success on a modified scale of the recent policy of the Lambeth Board of Guardians. A few months ago they opened at their infirmary a central drug store, from which the drugs required for their other institutions and for the needs of the outdoor poor are supplied. Dr. Baly, the medical superintendent, who has reported a very large increase in the price of drugs to the board, now informs the Guardians that a six months' supply of the most important is in hand. He has, however, given instructions that strict economy should be exercised in prescribing. The Guardians may feel extremely gratified that their foresight in establishing a central drug store will result not only in a considerable saving in expenditure, but also in an assured supply.

### GRAND STAND AS A HOSPITAL.

Consequent on the acceptance of the Epsom and Ewell War Hospital by the military authorities, the medical, administrative, and stretcher-carrying arrangements were ready by the 21st ultimo for the reception of the sick and wounded. The active workers in the scheme, of which Mr. C. P. Collier Jones and Mr. A. E. Williams are the hon. secretaries, were therefore expecting patients from the Royal Herbert Hospital, Woolwich, at the Grand Stand building. This is probably a unique site for a hospital, and is characteristic of the way in which emergency requirements have seized on every type of institution capable of adaptation to hospital purposes.

### NEW TREATMENT BATHS AT BATH.

The report of the baths development sub-committee has now been adopted for submission to Bath City Council. The proposals are, briefly, to expend £86,000 in the purchase of a site and the erection of new buildings covering some 20,440 feet super., which will incidentally open up additional Roman remains. The Queen's Bath and Inhalatorium are to receive alterations and a new lounge is to be added to the pump-room. The new buildings and alterations will provide an additional thirty-three bath-rooms and seventy-eight dressing-rooms, and the following new treatment baths:—Douche-massage, ladies' douche, moor baths, mud pack and Tyrnauer hot-air, as well as four-cell, high-frequency and static electrical treatment. This extension should be enough to show how genuine is the work done by home spas in competition with that previously appropriated by those on the Continent.

### THIS WEEK'S DRUG MARKET.

There is a perceptible improvement in the tone of the Drug market, and the prices of several articles show signs of receding, while in a few cases quotations are actually lower. This is to some extent due to the fact, noted last week, that goods are arriving from neutral countries and also in part to the circumstance that some holders seem to have underestimated the size of their stocks. Potassium, ammonium, and sodium bromides are distinctly lower in price. Acetyl-salicylic acid, which is now being made in this country, is also somewhat lower, and the same applies to salicylic acid and sodium salicylate. Antipyrine, acetanilide, and cocaine are also slightly lower in price. Cod-liver oil is practically unchanged, but the course of this market depends upon the situation in the North Sea. In the quinine market there is no change. There is a lower tendency in the prices of citric acid and tartaric acid. Opium, morphine, and codeine are unchanged. In a general sense it may be said that more inclination to do business is being evinced both by buyers and sellers.

